# INTEnsive care bundle with blood pressure reduction in acute cerebral hemorrhage trial (INTERACT3): study protocol for a pragmatic stepped-wedge cluster-randomized controlled trial

**DOI:** 10.1186/s13063-021-05881-7

**Published:** 2021-12-20

**Authors:** Lili Song, Xin Hu, Lu Ma, Xiaoying Chen, Menglu Ouyang, Laurent Billot, Qiang Li, Paula Muñoz-Venturelli, Carlos Abanto, Octavio Marques Pontes-Neto, Arauz Antonio, Mohammad Wasay, Asita de Silva, Nguyen Huy Thang, Jeyaraj Durai Pandian, Kolawole Wasiu Wahab, Chao You, Craig S. Anderson, for the INTERACT3 investigators

**Affiliations:** 1 The George Institute China, Peking University Health Science Center, Room 011, Unit 2, Tayuan Diplomatic Office Building, No. 14 Liangmahe Nan Lu, Chaoyang District, Beijing, China; 2 The George Institute for Global Health, Faculty of Medicine, UNSW, Level 5, King Street, Newtown, NSW 2042 Australia; 3 Department of Neurosurgery, West China Hospital, Sichuan University, NO.43, St. Guoxuexiang, Chengdu, China; 4 Clinical Research Center, Faculty of Medicine, Clinica Alemana Universidad del Desarrollo, Santiago, Chile; 5 The Cerebrovascular Disease Research Center, National Institute of Neurological Sciences, Cercado de Lima, Peru; 6 Department of Neurology, Ribeirão Preto Medical School, University of São Paulo, São Paulo, Brazil; 7 Instituto Nacional de Neurologia y Neurocirugia Manuel Velasco Suarez, Mexico City, Mexico; 8 Department of Medicine, The Aga Khan University, Karachi, Pakistan; 9 Clinical Trials Unit, Faculty of Medicine, University of Kelaniya, Colombo, Sri Lanka; 10 Stroke Unit, 115 Hospital, Ho Chi Minh city, Vietnam; 11 Neurology, Christian Medical College and Hospital, Ludhiana, India; 12 Department of Medicine, University of Ilorin & University of Ilorin Teaching Hospital, Ilorin, Nigeria; 13 Sydney Medical School, University of Sydney, Sydney, Australia; 14 Department of Neurology, Royal Prince Alfred Hospital, Sydney, Australia

**Keywords:** Stepped-wedge cluster-randomized trial, Clinical trial, Care bundle, management, Intracerebral hemorrhage, Stroke

## Abstract

**Background:**

Early intensive blood pressure (BP) lowering remains the most promising treatment for acute intracerebral hemorrhage (ICH), despite discordant results between clinical trials and potential variation in the treatment effects by approach to control BP. As the third in a series of clinical trials on this topic, the INTEnsive care bundle with blood pressure Reduction in Acute Cerebral hemorrhage Trial (INTERACT3) aims to determine the effectiveness of a goal-directed care bundle protocol of early physiological control (intensive BP lowering, glycemic control, and pyrexia treatment) and reversal of anticoagulation, in acute ICH.

**Methods:**

INTERACT3 is a pragmatic, international, multicenter, stepped-wedge (4 phases/3 steps), cluster-randomized controlled trial to determine the effectiveness of a multifaceted care package in adult (age ≥ 18 years) patients (target 8360) with acute ICH (< 6 h of onset) recruited from 110 hospitals (average of 19 consecutive patients per phase) in low- and middle-income countries. After a control phase, each hospital implements the intervention (intensive BP lowering, target systolic < 140 mmHg; glucose control, target 6.1–7.8 mmol/L and 7.8–10.0 mmol/L in those without and with diabetes mellitus, respectively; anti-pyrexia treatment to target body temperature ≤ 37.5 °C; and reversal of anticoagulation, target international normalized ratio < 1.5 within 1 h). Information will be obtained on demographic and baseline clinical characteristics, in-hospital management, and 7-day outcomes. Central trained blinded assessors will conduct telephone interviews to assess physical function and health-related quality of life at 6 months. The primary outcome is the modified Rankin scale (mRS) at 6 months analyzed using ordinal logistic regression. The sample size of 8360 subjects provides 90% power (*α* = 0.05) to detect a 5.6% absolute improvement (shift) in the primary outcome of the intervention versus control standard care, with various assumptions.

**Discussion:**

As the largest clinical trial in acute ICH, INTERACT3 is on schedule to provide an assessment of the effectiveness of a widely applicable goal-directed care bundle for a serious condition in which a clearly proven treatment has yet to be established.

**Trial registration:**

ClinicalTrials.gov NCT03209258. Registered on 1 July 2017. Chinese Trial Registry ChiCTR-IOC-17011787. Registered on 28 June 2017

**Supplementary Information:**

The online version contains supplementary material available at 10.1186/s13063-021-05881-7.

## Administrative information

Note: the numbers in curly brackets in this protocol refer to SPIRIT checklist item numbers. The order of the items has been modified to group similar items (see http://www.equator-network.org/reporting-guidelines/spirit-2727-statement-defining-standard-protocol-items-for-clinical-trials/).

**Table:** 

Title {1}	INTEnsive care bundle with blood pressure Reduction in Acute Cerebral Hemorrhage trial (INTERACT3): study protocol for a pragmatic stepped-wedge cluster-randomized controlled trial
**Trial registration {2a and 2b}**	ClinicalTrials.gov identifier: NCT03209258. Chinese Trial Registry identifier ChiCTR-IOC-17011787.
**Protocol version {3}**	Version 3.0 – 12 August 2019
**Funding {4}**	MRC Joint Global Health Trials Call 9 (MR/T005009/1), National Health and Medical Research Council (NHMRC) of Australia (APP1149987), the West China Hospital Outstanding Discipline Development 1-3-5 Program (ZY2016102), and Sichuan Credit Pharmaceutical Co., Ltd, and Takeda (China) International Trading Co., Ltd
**Author details {5a}**	^1^The George Institute China, Peking University Health Science Center, China ^2^The George Institute for Global Health, Faculty of Medicine, UNSW, Australia ^3^West China Hospital, Sichuan University, China ^4^Faculty of Medicine Clínica Alemana Universidad del Desarrollo, Chile ^5^National Institute of Neurological Sciences, Peru ^6^University of São Paulo, Brazil ^7^Instituto Nacional de Neurologia y Neurocirugia Manuel Velasco Suarez, Mexico ^8^The Aga Khan University, Pakistan ^9^University of Kelaniya, Colombo, Sri Lanka ^10^115 Hospital, Vietnam ^11^Chiristian Medical College and Hospital, India ^12^University of Ilorin & University of Ilorin Teaching Hospital, Nigeria ^13^University of Sydney, Australia ^14^Royal Prince Alfred Hospital, Australia
**Name and contact information for the trial sponsor {5b}**	The George Institute for Global Health (Australia) Beijing Representative Office, Room 011, Unit 2, Tayuan Diplomatic Office Building, No. 14 Liangmahe Nan Lu, Chaoyang District, Beijing, China. Phone: + 86 10 8280 0577; Fax: +86 10 8280 0177; Email: interact3@georgeinstitute.org.cn
**Role of sponsor and funder {5c}**	The study sponsors and funders had no role in the design, execution, analyses, interpretation of data, or decision to submit results for this study

## Introduction

### Background and rationale {6a}

Acute spontaneous intracerebral hemorrhage (ICH) is the most severe type of stroke, for which two thirds of patients either die or are disabled, and where the overall rates and outcomes have remained relatively stable in recent decades [1, 2]. Although ICH accounts for approximately 10–20% of strokes in high-income countries, proportional frequencies and rates are much higher in low- and middle-income countries (LMICs) causing considerable personal, social, and economic burden in large high-risk populations [3–5].

Although slow progress has been made in reliably establishing effective medical (and surgical) treatments for ICH, intensive blood pressure (BP) lowering is the most attractive as it is relatively low-cost, widely applicable, and supported by strong epidemiological data for the frequency and prognostic significance of hypertension in ICH [6]. This treatment appears safe and effective when given early after the onset of ICH [7] when most of the bleeding occurs, defined as hematoma growth or expansion [8]. Thus, BP lowering in the context of other active, supported, and organized care [9, 10] could provide large absolute benefits even if the benefits to individual patients are modest. Yet, there are ongoing concerns over the size of potential benefits and harms [11] across different types of patients, which influence guideline recommendations and the unifying of clinical practice [12]. There is also uncertainty over the benefits of controlling other abnormal physiological variables, such as hyperglycemia [13, 14] and pyrexia [15], as well in the reversal of anticoagulation [16], which are associated with hematoma growth and adverse outcomes [17, 18], but where there are considerable challenges to providing the necessary evidence from adequately powered randomized studies.

Clustering interventions together as part of a care bundle have shown encouraging results. For example, the Australian Quality in Acute Stroke Care (QASC) cluster trial [19] showed that a protocol to manage fever, hyperglycemia, and assessments of swallowing function improved recovery for patients with acute stroke, while a quality improvement “before-and-after” evaluation of protocol involving anticoagulation reversal, intensive BP lowering, and rapid triage and access to neurosurgery and critical care showed improved survival in patients with ICH admitted to hospitals in Greater Manchester, UK [20]. Our analyses of the second phase of INTEnsive blood pressure Reduction in Acute Cerebral hemorrhage Trial (INTERACT2) showed that the scoring of abnormal baseline variables covering BP, glucose, body temperature, and prior use of anticoagulants independently predicted poor outcome in ICH [21].

We therefore initiated the third INTEnsive care bundle with blood pressure Reduction in Acute Cerebral hemorrhage Trial (INTERACT3) with collaborators in 10 Asian, Latin American, and African countries (Appendix 1) to determine the effectiveness of a goal-directed care bundle of active management involving early physiological control (intensive BP lowering, glycemic control, and pyrexia treatment) and reversal of anticoagulation, compared against usual standard of care. Using a pragmatic, one direction, stepped-wedge, cluster-randomized controlled trial design, the aims are to provide evidence of the applicability of a readily scalable, goal-directed protocol that could be widely applied in clinical practice, while also providing information on the effectiveness of particular components and across certain characteristics of patients. Herein, we report the final version of the trial protocol, compliant with the Standard Protocol Items: Recommendations for Interventional Trials (SPIRIT) reporting guideline (Appendix 2).

## Objectives {7}

### Hypothesis

A goal-directed care bundle of active management involving early physiological control (intensive BP lowering, glycemic control, and pyrexia treatment) and reversal of anticoagulation improves functional outcome after acute ICH.

### Research questions

1. Does a goal-directed care bundle of active management involving early physiological control (intensive BP lowering, glycemic control, and pyrexia treatment) and reversal of anticoagulation improve functional outcome after acute ICH compared to usual standard of care (the null hypothesis is that there is no difference in functional outcomes between treatment groups)?
2. Does a care bundle improve other clinical outcome measures of death and dependency, separately on death and physical function, health-related quality of life (HRQoL), days of hospitalization, and living circumstances, compared to usual care?
3. Does a care bundle reduce hematoma growth and perihematomal edema?
4. Is there heterogeneity in the treatment effects across particular patient characteristics?
5. Are there clinical benefits associated with the separate components of the care bundle?
6. What factors support or impede the integration of a care bundle protocol for ICH in hospital practice?
7. What are the costs of implementing the care bundle?

## Trial design {8}

**Fig. 1:**
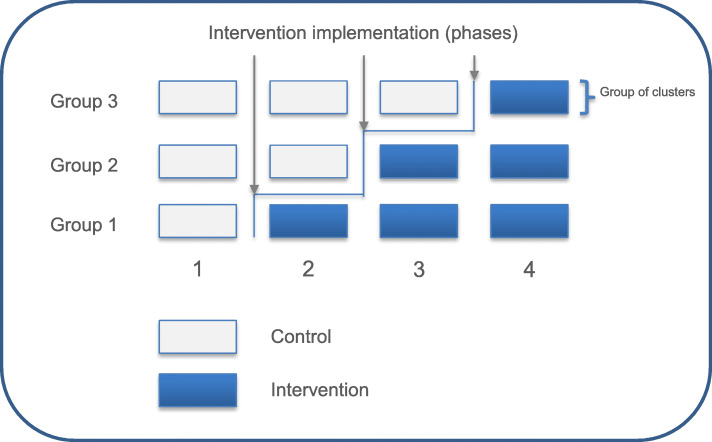
Stepped-wedge design

**Fig. 2:**
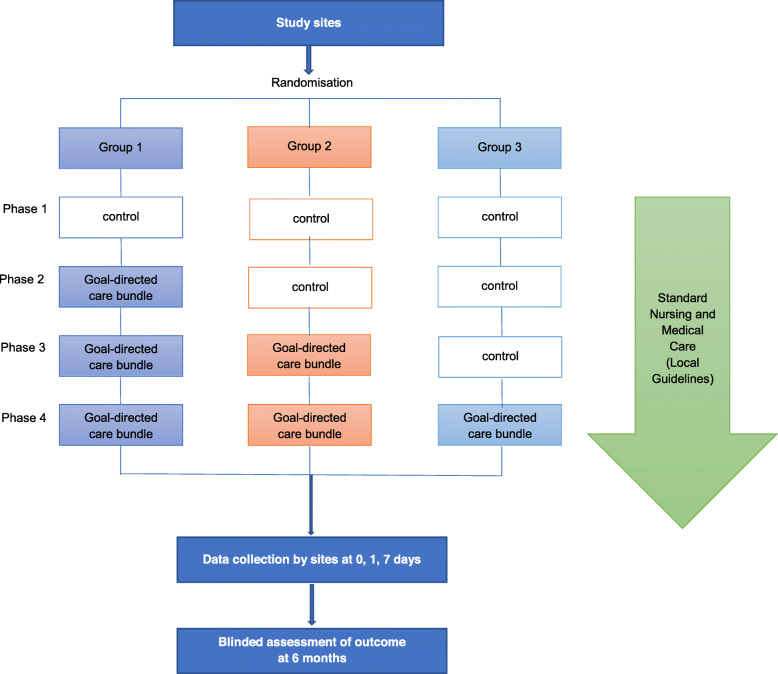
Study schema

The INTERACT3 study is an international, multicenter, prospective, stepped-wedge, cluster-randomized controlled, blinded outcome assessed (PROBE) trial, which is being conducted through a network of investigators in LMICs. After participating hospital sites commence in a usual care “control” phase, they progressively transfer to the intervention (care bundle) at pre-determined stages through random allocation into 3 groups (Fig. 1). In each phase, hospitals aim to reach an average target of 19 consecutive ICH patients who fulfill the eligibility criteria, but this number may vary from 1 to 50 patients according to service configuration and patient volumes. However, each phase has a 3-month time limit, to ensure that the intervention period of the study is completed over 12 months. The study design is summarized in Fig. 2.

## Methods: participants, interventions, and outcomes

### Study setting {9}

The trial is being conducted in approximately 110 hospitals in 9 LMICs: Brazil, China, India, Mexico, Nigeria, Pakistan, Peru, Sri Lanka, and Vietnam, and one high-income country, Chile.

### Eligibility criteria {10}

#### Hospital sites

Hospitals are eligible if they do not have any organizational protocols for the management of ICH, or if they use protocols different to those proposed in the study and are comfortable about switching to the proposed interventional bundle. All patients with acute ICH who present to participating sites are included on a register during the study period, with those fulfilling the eligibility criteria included in the study. Brief details of all patients with acute ICH who are screened but not included, as well as those recruited, are recorded on the screening/enrolment form. All eligible patients are provided with an approved patient information sheet (PIS) or patient responsible information sheet (PRIS) and consent form (CF). A participant is anyone for whom consent is obtained for permission to collect their medical and personal information, and to be contacted at 6 months for a follow-up assessment of their health status.

### Study patient characteristics

Patients are eligible to participate if they:

- Are an adult (aged ≥18 years)
- Have an acute stroke syndrome due to presumed spontaneous ICH, defined as the sudden occurrence of bleeding into the parenchyma of the brain that may extend into the ventricles and, in rare cases, into the subarachnoid space, confirmed by clinical history and on CT brain imaging
- Present within 6 hours of the onset of symptoms

Patients with an ICH that is presumed secondary to a medical treatment, for example antithrombotic or anticoagulation therapy, are eligible, but those that follow treatment with intravenous thrombolysis or endovascular thrombectomy for acute ischemic stroke are ineligible. If the precise timing of the first onset of symptoms or signs of the qualifying event are unknown, then the time of onset is taken as the last time at which the patient was known to be well.

Patients are excluded if they:

- Have definite evidence that the ICH is secondary to a structural abnormality in the brain (e.g., an arteriovenous malformation [AVM], intracranial aneurysm, tumor, trauma, or previous cerebral infarction)
- Have had recent thrombolysis/thrombectomy
- Have a high likelihood that the patient will not adhere to the study treatment and/or follow-up regimen

In each case, the decision about a patient’s eligibility is based upon the attending clinician’s interpretation of these eligibility criteria.

### Consent process {26a}

Each participating site must obtain written approval(s) from their hospital research ethics committee (EC) (e.g., institutional review board [IRB]), and any other relevant regional or national body, before patient recruitment commences (Appendix 3). A variable, mixed consent process is used, according to local/national rules and regulations:

- Cluster guardian consent or appropriate approval (e.g., signed by the General Manager or Chief Executive of the hospital, or Head of Neurology/Neurosurgery/Stroke Department) for patients to receive the randomized care bundle to be implemented for patients acute ICH in the Emergency Department, Stroke Unit, Intensive Care Unit, or Neurology/Neurosurgery Wards
- Individual standard consent for the collection of data through in-person assessment and data extraction from medical records during the hospital stay and follow-up, and for release of personalized information for research purposes to allow centralized follow-up at 6 months following admission

### Additional consent provisions for collection and use of participant data and biological specimens {26b}

The study consent process includes permission for additional analysis of the collected data for systematic reviews and individual patient data pooling projects. In a subset of 1000 consecutive patients across sites, all brain imaging over a 7–10-day period are collected and uploaded in Digital Imaging and Communications in Medicine (DICOM) format for analysis of the characteristics of the ICH and underlying brain structure at a core imaging laboratory at The George Institute for Global Health (TGI), Sydney, Australia. No biological specimens are collected.

### Interventions

#### Explanation of the choice of comparators {6b}

The active comparator (intervention) is a goal-directed care bundle that involves rapid correction (< 1 h) in those participants who have abnormal physiological and hematological variables during their in-hospital stay. The intervention includes early intensive BP lowering, glucose control, treatment of pyrexia, and reversal of previous use of anticoagulants. Sites without a formal institutional protocol on how to control BP, glucose, fever, and previous anticoagulants usage in acute ICH stage, or their protocol differentiated from the protocol provided in this study were eligible. Each site will keep their usual care or previous routine protocol before transferring to intervention phase. The usual care phase in place before switching to the intervention will act as the control comparator. The intention of introducing this intervention bundle is to enhance the implementation of guidelines or recommendations, narrow the gap between evidence and practice in LMICs, and improve outcome for patients with acute ICH. A one-direction stepped-wedge cluster design was used to allow all hospitals to change to improved systems of care.

#### Intervention description {11a}

The care bundle involves one or more components according to whether a participant has abnormal physiological or hematological parameters, as outlined below:

- *Early intensive BP lowering*, where the goal is to achieve a target systolic BP level of < 140 mmHg within 1 h of initiation, and to maintain this level for the next 7 days, or hospital discharge should this occur earlier. Intravenous BP lowering is the preferred treatment to commence as soon as possible upon the patient’s admission. It is expected that intravenous therapy will continue to be required while any oral antihypertensive therapy is initiated, in order to maintain smooth BP control. A systolic BP of 130 mmHg is considered the lower threshold in which the treatment is to cease. Each site receives a standardized, stepped titratable, intravenous BP lowering protocol, based on available medications, that is established in advance.
- *Intensive glucose control*, where the goal is to achieve a target blood glucose level of 6.1–7.8 mmol/L and 7.8–10.0 mmol/L for without and with diabetes mellitus, respectively, and to maintain this level for the next 7 days, or hospital discharge should this occur earlier. In the intensive-treatment phase, a continuous infusion of insulin (50 international units [IU] in 50 mL of 0.9% sodium chloride for use in a pump) is recommended to be started as soon as possible if the blood glucose level exceeds 7.8 mmol/L and 10 mmol/L according to non-diabetes or diabetes, respectively. Adjustments of the insulin dose are based on measurements of whole-blood glucose, performed at 1- to 4-hourly intervals, with the aid of a glucose analyzer. Patients can be fed continuously with intravenous glucose at the time of admission, but total parenteral, combined parenteral and enteral, or total enteral, feeding is instituted the following day according to a standardized schedule whereby 20–30 non-protein kilocalories per kilogram of body weight per 24 h and a balanced composition (including 0.13 to 0.26 g of nitrogen per kilogram per 24 h, and 20 to 40% of nonprotein calories in the form of lipids) is achieved as early as possible.
- *Treatment of pyrexia*, where the goal is to achieve a body temperature level < 37.5 °C within 1 h of initiation and to maintain this level for the next 7 days, or hospital discharge should this occur earlier. Measurement of temperature is according to local practice. Patients allocated to the intensive group are to receive measures of their body temperature every 4 h over 72 h after admission. Patients with an increase (≥37.5 °C) in body temperature are to commence treatment as soon as possible, according to a standardized anti-pyrexia treatment protocol, based on available medications established in advance.
- *Reversal of anticoagulation*, where the goal is to achieve an international normalized ratio (INR) of < 1.5 within 1 h of treatment, and to maintain this level for the next 7 days, or hospital discharge should this occur earlier. All patients with ICH are to have a check of their blood INR immediately upon presentation. Those with an elevated INR (> 1.5) who are allocated to the intensive group should receive within 1 h of diagnosis: 5–10 mg of vitamin K administered slowly intravenously; and according to availability in the hospital, either intravenous fresh frozen plasma (FFP) at 20 mL/kg, after blood group typing or by using AB group plasma supplied by local transfusion units; or 30 IU/kg of intravenous four-factor prothrombin complex concentrate (PCC). The speed of the infusion of FFP or PCC should be as fast as the condition of the patient allows. Patients with an INR > 1.5 at 3 h after the start of treatment are to receive PCC (if INR ≤2.0, 10 IU/kg; if INR > 2.0, 30 IU/kg) as rescue treatment.

#### Criteria for discontinuing or modifying allocated interventions {11b}

All hospitals are randomly assigned to the intervention and encouraged to implement the intervention protocol as completely as possible. However, they may modify sections or skip them completely, according to clinical reasons.

#### Strategies to improve adherence to the intervention {11c}

The intervention protocol is given to investigators only after completion of control phase. An online training is organized for each site before starting the intervention. Regular intervention quality reports are provided to each site during intervention phase, and there is remote communication and on-site monitoring to improve the adherence to the intervention. Moreover, a process evaluation, designed to gain insights into the barriers and facilitators to change systems of care and implementation of the protocol, is undertaken through formative stakeholder engagement interviews during the course of the study.

#### Relevant concomitant care permitted or prohibited during the trial {11d}

Medical or surgical treatments besides those required within the care bundle are permitted during the study, but they need to be recorded in the case report form (CRF).

#### Provisions for post-trial care {30}

Not applicable. This study is evaluating a quality improvement protocol for use of interventions that are already available in routine practice which can potentially be sustained beyond completion of the study.

### Outcomes {12}

The primary outcome is functional recovery according to the modified Rankin scale (mRS) measured at 6 months and analyzed as an ordinal outcome (shift across all scoring categories of physical function that range from 0 to 5, and death as 6) [22].

Secondary outcomes include death or neurological deterioration according to a change in scores on the National Institute of Health stroke scale (NIHSS) [23] at 7 days, and poor outcome (defined by mRS scores 3–6), separately on death and disability (mRS scores 3–5), HRQoL using the EuroQoL Group 5-Dimension self-report questionnaire (EQ-5D) [24], duration of hospital stay, and residence, all measured at 6 months.

The safety outcomes of any all-cause and cause-specific serious adverse events (SAEs) are recorded according to standard definitions for the duration of follow-up.

### Participant timeline {13}

The schedule of randomization for sites as well as enrolment, treatment allocation, and assessments for participants is outlined in the Table 1 and Fig. 2.

**Table 1:** Assessment schedule

Evaluation	Screen/enrolment log	Baseline	Day 1	72-h monitoring chart	7 days or hospital separation (discharge, transfer, or death)	6-month follow-up
**Forms**	**A**	**B**	**C**	**D/E**	**F**	**G**
Screen	X					
Eligibility	X					
Consent/re-consent	X					
Contact details	X					
Vital signs	X	X	X		X	
Physical parameters				X		
GCS		X				
NIHSS		X			X	
Medical history		X				
mRS					X	X
EQ-5D						X
Routine blood tests		X			X	
Brain imaging		X	X		X	
Standard stroke care		X			X	
Final diagnosis					X	
Medications in use			X	X	X	X
SAEs			X		X	X

*EQ-5D* EuroQoL Group 5-Dimension self-report questionnaire, *GCS* Glasgow Coma Scale, *mRS* modified Rankin scale, *NIHSS* National Institutes of Health stroke scale, *SAEs* serious adverse events

### Sample size {14}

The study is designed with 90% power (*α* = 0.05) to detect a 20% reduction in the odds (common odds ratio of 0.80) of worse functional outcome using ordinal logistic regression. Assuming a distribution of mRS in the usual care control arm that is similar to that observed in the standard BP control arm of the INTERACT2 study [7] (i.e., 7.6%, 18.0%, 18.8%, 16.6%, 19.0%, 8.0%, and 12.0% for mRS scores of 0 to 6, respectively), this corresponds to a 5.6% absolute improvement in the proportion of patients experiencing a poor outcome (mRS scores 3-6), that is from 55.6% down to 50%. This also translates into a 10% relative risk reduction (relative risk of 0.90). The combination of interventions as part of an intensive care bundle is assumed to provide a greater treatment effect than the use of BP lowering alone (in INTERACT2, the treatment effect was 4% absolute).

The study plans to recruit 110 sites in a stepped-wedge design consisting of 3 groups and 4 phases. Each group would, therefore, include approximately 36 sites. Assuming an intraclass correlation coefficient (ICC) of 0.044, which is similar to that of the INTERACT2 [7] and Head Position in Acute Stroke Trial (HeadPoST) [25] studies that included large numbers of hospitals in China, each site would be required to recruit an average of 18 patients per phase. Accounting for 5% of participants with a missing outcome, each site would need to target an average of 19 patients per phase, thus leading to a total sample size of 8360 patients (110 sites × 4 phases × 19 patients).

We recognize there will be variability in the number of patients who will be recruited at each site, with very large hospitals recruiting as many as 50 patients per phase while smaller hospitals may only recruit a few patients per phase. If the sample size was inflated to accommodate this issue as has been used in parallel cluster trials [26] (e.g., using an inflation factor of approximately 1.3), the sample size would need to reflect 25 patients per site per phase (i.e., 11,000 patients in total). However, given that this adjustment would be a very conservative scenario and that the effect of variability in cluster sizes is expected to be mostly mitigated by the randomization process, including stratification by size [27], we have elected to maintain the target average of 19 patients per site per phase for a total sample size of 8360 patients. Assuming a worst-case scenario for the effect of cluster size variability on power, this sample size would still provide at least 80% power to determine the proposed treatment effect.

### Recruitment {15}

All eligible ICH patients who present to each participating hospital from the start date are prospectively and consecutively enrolled into the study.

## Assignment of intervention: allocation

### Sequence generation {16a}

The unit of randomization is the hospital site, randomly assigned by a statistician not otherwise involved in the study using a pre-specified randomization schedule with permuted blocks. Participating sites are stratified according to country and the estimated recruitment capacity (ranging from 40, 80, 120, 160, and 200).

### Concealment mechanism {16b}

A statistician not otherwise involved in the study will randomly allocate hospitals into 3 groups, with each group to assign the time of switching from usual care (control) to care bundle (intervention) across hospital sites. The randomization group will be notified to the site within 2 weeks of the agreed date of commencing the phase of study. All eligible ICH patients presenting to participating hospitals from the start date will be prospectively and consecutively enrolled, and when assigned to the intervention phase will be managed with the care bundle as usual clinical practice.

### Implementation {16c}

In phase 1, all hospitals will be observed under usual care “control” conditions according to usual management of ICH patients. In phase 2, the first cluster of hospitals (group 1) will start implementing the intervention (care bundle), and then sequentially, groups 2 and 3 will start implementing the interventional package in phases 3 and 4, respectively, so that by phase 4, all hospitals will be receiving the intervention, with those in group 1 having the intervention for longest and those in group 3, the shortest. Once the site has completed the necessary control usual care observational period, they will receive training on the application of the care bundle intervention during the study. The site investigator will be informed to pause enrolment when the patient number or study duration in control phase has reached the pre-specified target, and then will be instructed to transfer over to the intervention phase after a recruitment interval of 7–10 days.

## Assignment of interventions: blinding

### Who will be blinded {17a}

The treatment is open label to both investigators and patients at sites. All other investigators, statisticians, and endpoint assessors are blind to the treatment allocation, who are trained to collect outcome measures by telephone at 6 months.

### Procedures for unblinding if needed {17b}

Not applicable. This study is an unblinded, hospital-level intervention.

## Data collection and management

### Plans for assessment and collection of outcomes {18a}

Hospitals are required to collect data on participating patients at admission (baseline), 72 h monitoring chart, separation (day 1; day 7 or at discharge if earlier, transfer from the hospital or death), and all SAEs including death until the 6 months of follow-up. The 6-month assessments will be conducted by an appropriately trained independent outcome assessor, using an assigned telephone script and kept blind to the treatment allocation. The assessor will be managed by the regional office in each country.

Table 1 illustrates the schedule and nature of the data collection required during the study period.

### Plans to promote participant retention and complete follow-up {18b}

The centralized follow-up assessment is anticipated to be more challenging than in conventional individual-patient clinical trials because the study uses broad inclusion criteria and the consecutive recruitment of patients; there is a high “floating” population in LMICs where people often change their mobile telephone numbers; and there is a relatively long interval between the time of discharge from hospital and follow-up (6 months). Several strategies are used to ensure that the rate of lost-to-follow-up is kept low. First, investigators emphasize to participating patients (and their responsible person[s]) during the consent process that they are to receive a telephone call from a person in a centralized office to check on their health status at 6 months. Second, the telephone number of the central office is provided on a small card to patients and/or their relatives at the time of discharge from hospital as a reminder to accept the follow-up telephone call. Third, investigators collect a range of contact information, including those of the patient and of several relatives and/or friends. Finally, the investigator will try to reassure patients and relatives of the telephone number to overcome any mistrust of unfamiliar telephone calls.

### Data management {19}

Hospital sites receive paper versions of the CRFs and a procedure manual to serve as a reference guide in using the database; each data element is defined to ensure investigators are accurate and consistent in data entry. All data entry is completed using a password-protected Internet-based data management system which allows individual log-in; all investigators and coordinating/monitoring research staff are required to ensure security, privacy and confidentiality. Only research staff listed on site delegation logs are given access to the data management system, which allows real time data entry and the generation of queries for values entered outside of valid ranges, and for consistency checking. All computerized forms are electronically signed (by use of the unique password) by authorized study staff; all changes made following the initial entry have an electronic dated audit trail.

### Confidentiality {27}

Every precaution is taken to respect the privacy of participants in the conduct of the study. Only de-identified data will be used for statistical analysis and the publication of results to maintain confidentiality. However, as a part of the centralized follow-up service, the International Coordinating Center (ICC) at TGI and Regional Coordinating Centers (RCCs) will use contact sources recorded by the hospital sites. Only name, telephone numbers, next of kin, and primary medical practitioner contact details (if applicable) are sent to the follow-up center for the follow-up assessments. The information is encrypted and password-protected using an MS Excel lock form before being sent by email in batches. This information is included in the PIS and PRIS. In the course of monitoring for data quality and adherence to the study protocol, research staff will refer to source documents (medical records) at participating hospitals. This information is also included in the PIS and PRIS. All individual and site information will be de-identified in reports and results to further protect the confidentiality of participants.

### Plans for collection, laboratory evaluation, and storage of biological specimens for genetic or molecular analysis in this trial/future use {33}

Not applicable. Biological specimens are not collected as part of this study.

## Statistics methods

### Statistical methods for primary and secondary outcomes {20a}

All analyses will be undertaken at the patient level on an intention-to-treat (ITT) basis at each site using generalized estimating equations (GEE) or random-effects regression to account for clustering. The primary outcome of a shift (improvement) in scores on the mRS at 6 months will be analyzed by means of an ordinal logistic regression, with mRS as a dependent variable with 7 levels (0 [no residual symptom] to 6 [death]). The model will include the trial phase (1 to 4), the randomized arm (control or intervention) corresponding to each phase, and the size of the site (as used in the stratification). The secondary outcome of neurological change at 7 days will be analyzed with the same method [28]. Binary and continuous secondary outcomes will be analyzed using a similar approach but using logistic and linear regression, respectively.

All analyses will be adjusted for clustering within center, hospital size (stratification variable), and for trial phase. In addition, sensitivity analyses will be conducted after considering potential prognostic variables as well as the effect of time to adjust for potential background secular trends, including the potential impact of the COVID-19 pandemic.

No adjustment for multiplicity is planned as there are only a small number of pre-specified efficacy outcomes being investigated. A detailed statistical analysis plan will be finalized before unblinding and database locked and posted on a pre-print server.

### Interim analyses {21b}

Two “formal interim analysis” meetings will be held by Data and Safety Monitoring Board (DSMB) by teleconference (or face-to-face, if possible) to review data relating to treatment efficacy, patient safety, and quality of trial conduct.

A recommendation to discontinue INTERACT3 prematurely will be based upon there being clear evidence that the treatment provides protection or causes harm for an important clinical outcome.

The DSMB will work on the principle that a difference of at least 3 standard errors in an interim analysis of a major outcome event (e.g., death from all causes or independent survival at 6 months) between patients allocated to the intensive or the control group, to justify halting, or modifying the study, before the planned completion of recruitment. Given the minimal impact of this approach on the type-I error rate, no adjustment is made to the final significance level [29].

### Methods for additional analyses {20b}

#### Substudy—effects of treatment of hematoma growth in ICH

The effects of treatment on hematoma expansion and other indices including perihematomal edema will be evaluated in a subsample of 1000 patients (the earliest 7 recruitments in each of the control and intensive groups, respectively, for each site). Apart from the baseline CT scan, repeat CT scans (24 ± 3 h and 7 days) are required. CT imaging will be conducted according to standardized techniques and uploaded to purpose-built server, either directly from the hospital site (if they have suitable broadband internet) or via the RCC office. The LCC will keep a hard copy in an uncompressed DICOM format onto a CD-ROM for monitor site verification. The imaging data will be analyzed centrally by experts who will be kept blind to the treatment allocation. The primary efficacy measure is proportional absolute and relative changes (“growth”) in hematoma and perihematomal volumes according to standard measures at 24 h. Background measures of cerebral small vessel disease and “brain frailty” will also be recorded. Clinical outcomes are assessed over 6 months.

#### Process evaluation

In order to explore how the care bundle, a complex intervention, is implemented, as well as to understand clinicians’ perspectives, a prospective process evaluation will be conducted alongside the roll-out of the trial. Intervention fidelity, reach, dose, adoption, feasibility, and appropriateness of the goal-directed care bundle will be evaluated within the trial. Contextual conditions (current policies, settings resources, etc.) that may have impacted on the quality of the implementation are being assessed.

A mixed methods analytical approach is being used to address the objectives of process evaluation, with questions and indicators informed by the process evaluation framework of the Medical Research Council (MRC) [30]. The normalization process theory (NPT), which has been used for assessing how guidelines are embedded into routine practice, will serve as a conceptual framework to explore systematically how overall and for each of the interventions were adopted into patient care [31]. To assess the implementation and mechanism of impact of the interventions, semi-structured interviews and non-participants observations will be conducted among the primary implementers (physicians and nurses) using structured interview guides and observation templates. Focus group discussions will be conducted with project operation staff and site principal investigators to explore facilitators and barriers to the care bundle delivery by different stakeholders. Observational records, including hospital organization questionnaires, routine monitoring data, field notes, and CRFs, will also be used to triangulate qualitative findings to assess implementation quality, acceptability of the care bundle, and contextual factors, which may have impacted on outcomes. Sites involved in the process evaluations will be determined by purposive sampling in accordance with the pre-specified criteria to achieve representativeness. Results of the process evaluation will be used to monitor and document project implementation, as well as identify barriers, which the coordinating team uses to address in a timely fashion, such as increasing the confidence of clinicians in managing the intervention by providing them with additional training.

### Methods in analysis to handle protocol non-adherence and any statistical methods to handle missing data {20c}

All analyses including the primary analysis, will be conducted on the ITT population and using all available data. If more than 5% of patients have a missing primary outcome, we will use multiple imputations for sensitivity analysis.

### Plans to give access to the full protocol, participant-level data, and statistical code {31c}

The data collected is owned by the TSC. Datasets generated and/or analyzed will be available to all study investigators, and to investigators at other institutions around the world, according to a data sharing agreement. Data sharing will be available from 12 months after publication of the main results. Investigators are to make a formal request for data sharing through the Global Research Committee (GRC) of TGI, and according to a data sharing policy (https://georgeinstitute.sharepoint.com/TGIPolicy/Data%20Sharing%20Policy.pdf). Access will be controlled by the Principal Investigators (PIs) with the approval of the TSC.

## Oversight and monitoring

### Composition of the coordinating center and TSC {5d}

#### TSC

The TSC (independent Chair) will comprise independent members, the principal investigators, national leaders, expert academic researchers, patient representatives, and an observer from Medical Research Council (MRC) and will be governed by a Charter (Appendix 4). The TSC is responsible for overseeing the execution of the study design, protocol, data collection, and analysis plans, as well as publications.

#### CCC

The CCC is based at TGI China, supported by project staff, and is responsible for the day-to-day management of the study, data and project management, committee coordination, assistance with ethics committee and regulatory applications, protocol and procedures for training of participating sites, overseeing of initiation visits and activation of participating centers, monitoring of data quality and adherence to protocol, applicable guidelines and regulations, preparation of study data for analysis, and publication.

### Composition of the data monitoring committee, its role, and reporting structure {21a}

#### DSMB

The DSMB is independent of the sponsor and responsible for reviewing the safety, ethics, and outcomes of the study. The DSMB is governed by a Charter outlining responsibilities, procedures, and confidentiality, and reviews the accumulating unblinded data at regular intervals (Appendix 5).

### SAE reporting and harms {22}

An SAE is defined according to standard convention as any untoward medical occurrence that results in any of the following: (i) results in death; (ii) is life threatening in the opinion of the Investigator (at the time of the event); (iii) requires admission to hospital or prolongation of an existing hospital stay; (iv) results in persistent or significant disability or incapacity; (v) results in congenital anomaly or birth defect; or (vi) is an important medical event in the opinion of the Investigator that is not immediately life-threatening and does not result in death or hospitalization but which may jeopardize the patient or may require intervention to prevent one of the other listed outcomes.

An SAE form is provided for recording details that includes the event diagnosis, classification of the event using standard definitions, the Investigator’s opinion on the causal relationship to the care bundle, and the timing of the event. The Investigator will be responsible for ensuring that details of investigations and outcomes of an SAE are finalized. The SAE should be documented in the medical records or patient file, and signed and dated by the investigator, for audit and monitoring. All SAEs are reviewed by a medical monitor assigned to the trial. Safety outcomes are reported to the presiding Ethics Committees in line with their requirements every 6 months, as well as for review by the DMSB at each meeting.

### Frequency and plans for auditing trial conduct {23}

There are no plans for auditing trial conduct.

### Plans for communicating important protocol amendments to relevant parties (e.g., trial participants, ethics committees) {25}

All protocol amendments are approved by the TSC and communicated to co-investigators, commercial partners, and national leaders, who are responsible for study conduct in their country. The amended protocol can be implemented only after review and approval by ethics committees.

### Dissemination plans {31a}

In addition to relevant reports developed in formats suitable for various stakeholders, the findings will be published in high impact journals, presented at national and international conferences on stroke, cardiovascular disease, and hypertension. A series of seminars will be held at the end of the study, across China and in other participating LMICs, targeting academics, researchers, clinicians, and local health officers. Discussion and debate will assist in integrating the results, whatever the findings, into clinical practice and to influence the decisions of guideline and policy makers. Patient representatives are invited to attend TSC meetings and are asked to review study materials to ensure that the findings are able to be disseminated in an understandable manner to a broader audience.

## Discussion

INTERACT3 was initiated as a vanguard “start-up” phase in China in December 2017 and subsequently expanded to other countries following receipt of MRC grant funding in late 2019 that provides the necessary resources to achieve the required sample size and strengthen the generalizability and impact of the results.

However, conduct of the study has been adversely impacted by COVID-19 since January 2020. This led to a decline in the recruitment of patients in China and postponement of site activation elsewhere. Although the pandemic was well controlled in China during 2020, most of the other participating countries have had ongoing restrictions imposed by COVID-19; even so, they are on schedule for activation and roll-out in early 2021.

There have been challenges to implementing the intensive care bundle and data collection, as some investigators in LMICs have limited research experience, lack familiarity with Good Clinical Practice requirements, and have busy workloads. Quality control performance metrics covering the intervention variables at study and site levels are reported to sites on a monthly basis. Efforts to address issues identified through monitoring and the process evaluation have been undertaken in training sessions at investigator meetings and teleconferences, and with individual site investigators. The learnings gained in China are used to inform quality control systems introduced in the other countries.

INTERACT3 is the largest clinical trial in ICH that can address ongoing uncertainties over the effectiveness of early intensive BP lowering as well as the utility of managing other abnormal physiological variables, rapid correction of anticoagulation, and more broadly of an active management protocol in this serious condition. The pragmatic cluster clinical trial design of INTERACT3 allows the recruitment of a broad range of patients, many of whom have large hematomas or require early surgical intervention that were purposely excluded from previous clinical trials of early BP lowering. The results of this study will inform the effectiveness of a widely applicable goal-directed care bundle in acute ICH.

## Trial status

The study has been approved by relevant ethics committees and regulatory bodies at country level and local sites in China, Chile, Peru, Pakistan, Brazil, Mexico, Vietnam, Sri Lanka, India, and Nigeria. According to funding request from MRC, an additional approval had been obtained from Research Ethics Committee (REC) of the University of Leicester, UK.

Patient enrolment commenced in December 2017 and is planned to end on 30 March 2022. As of 31 December 2020, 5917 participants have been enrolled at 93 sites from China, Chile, Peru, Brazil, and Pakistan. The current protocol is version 3.0, and all protocol updates have been approved by TSC and Ethics Committees and communicated with investigators and DSMB members.

## Supplementary Information

The online version contains supplementary material available at http://doi..

**Additional file 1.** List of INTERACT3 collaborators.

**Additional file 2.** SPIRIT checklist.

**Additional file 3.** Informed consent materials.

**Additional file 4.** TSC Charter.

**Additional file 5.** DSMB Charter.

## Data Availability

Datasets generated and/or analyzed for INTERACT3 will be available to all study investigators, and investigators from other institutions around the world, according to a strict data sharing agreement. Data sharing will be available from 12 months after publication of the main results. Investigators are to make a formal request for data sharing through the Research Office of The George Institute. Access will be controlled by the Principal Investigators, with the approval of the TSC.

## Abbreviations

BP: Blood pressure
CCC: Central coordinating center
CT: Computerized tomography
DSMB: Data and safety monitoring board
EC: Ethics committee
HeadPoST: Head position in acute stroke trial
ICH: Intracerebral hemorrhage
INR: International normalized ratio
INTERACT2: The second phase, INTEnsive blood pressure reduction in acute cerebral hemorrhage trial
INTERACT3: The third INTEnsive care bundle with blood pressure reduction in acute cerebral hemorrhage trial
IRB: Institutional review board
LMICs: Low- and middle-income countries
MRC: Medical research council
mRS: Modified rankin scale
NIHSS: National Institute of Health stroke scale
SAE: Serious adverse event
SAP: Statistics analysis plan
TSC: Trial steering committee
