# Supplementary material for: INTEnsive care bundle with blood pressure reduction in acute cerebral hemorrhage trial (INTERACT3): study protocol for a pragmatic stepped-wedge cluster-randomized controlled trial
Source: Trials. 2021 Dec 20;22:943. doi: 10.1186/s13063-021-05881-7 (PMC8686093; doi:10.1186/s13063-021-05881-7)
Supplement: Supplementary file 1 — Additional file 1. List of INTERACT3 collaborators. [file 13063_2021_5881_MOESM1_ESM.docx]

**List of INTERACT3 collaborators**

The INTERACT3 investigators and collaborators are as follow:

Trial Steering Committee: T. Robinson (Chair); J. Jaime Miranda (Deputy Chair); C.S. Anderson (Principal Investigator); C. You (Co-Principal Investigator); L. Song (Project leader); A. Parry-Jones; N. Sprigg; A. Bamford (consumer representative; O. Smith (consumer representative).

National Leaders: China – C. You; Chile – P. Muñoz-Venturelli; Peru – C. Abanto; Brazil – O. Marques Pontes-Neto; Mexico – A. Arauz; Pakistan – M. Wasay; Sri Lanka – A. de Silva, Vietnam – N. Huy Thang; India – J. D.Pandian; Nigeria – K. W. Wahab.

Data Safety and Monitoring Committees: R. Herbert (Chair); C. Chen; W. Whiteley; R. Hu.

Trial Statistician: L. Billot, Q. Li.

Data Management: Y. Ning.

Central Coordinating Centre (The George Institute, Sydney, Australia): L. Song, X. Chen, X. Hu, L. Ma, A. Malavera, R. Anderson, M. Ouyang, L. Hatchwell, Z. Meng.

Regional Coordinating Centres: China (The George Institute China): C. Zhang, G. Cheng, R. Luo, H. Shi, J. Cheng, Y. Dong, S. Wang, Y. Zhang, P. Yi; South America (Chile) and Mexico: P. Muñoz-Venturelli, F. Gonzalez, F. Urrutia; Pakistan: M. Wasay, A. Anjum, D. Begum; Sri Lanka: A. de Silva, P. Ellawalla, S. Weerawardena, S. Budurdeen, M. Warangi; Nigeria: K. W. Wahab, S. Adeniyi; India: J. D. Pandian, M. Khanna, D. Arora; Vietnam: N. Huy Thang, U. Hong; L. Le, H. Thi Thuy Duong, M. Yen.

Site Principal Investigators (according to country and centre): China -West China Hospital Sichuan University: C. You; Zigong Fourth People’s Hospital: M. Chen; The First People’s Hospital of Yibin: X. Cao; Suining Central Hospital: Y. Jiang; Nanchong Central Hospital: J. He; Mianyang Central Hospital: Z. Li; Dazhu County People’s Hospital: S. Zhu; Chongzhou People’s Hospital: Y. Li; Yaan People’s Hospital: J. Wu; The Second People’s Hospital of Yibin: Y. Yi; The First People’s Hospital of Neijiang : C. Liu; The Second People’s Hospital of Neijiang: M. Zhang; Mianzhu People’s Hospital: P. Wei; Sichuan Mianyang 404 Hospital: W. Xiao; Guangyuan Central Hospital: D. Zhong; Dujiangyan People’s Hospital: Z. Zhou; Dayi County People’s Hospital: C. Yang; The First People's Hospital of Shuangliu District, Chengdu: J. Lei; Chengdu Second People’s Hospital: X. Xu; Sichuan Provincial People’s Hospital: H. Tan; Santai County People’s Hospital: X. Tang; West China Longquan Hospital Sichuan University: F. Chen; Pangang Group General Hospital: J. Wang; Anshun People’s Hospital: Z. Rao; The People’s Hospital of Bishan Chongqing: K. Mu; Chongqing Traditional Chinese Medicine Hospital: C. Luo; Affiliated Hospital of North Sichuan Medical College: X. Tang; The First Affiliated Hospital of Chengdu Medical College: J. Yang; Chongqing Emergency Medical Centre: X. Hu, Chengdu Fifth People’s Hospital: JS. LiuLuo; Panzhihua Central Hospital: J. Wei; Qiandongnanzhou People's Hospital: H. Yu; The First Hospital of Kuming: J. Liu; The First People's Hospital of Yunnan Province: J. Zhao; The People's Hospital of Leshan: N. Zheng; The Third People’s Hospital of Chengdu: YS. FanZhang; People's Hospital of Deyang City: F. Ye; The Third Hospital of Mianyang: Y. Liu; The First People's Hospital of Yiliang: P. Wang; Peking University Third Hospital Yanqing Hospital : T. Liu; The Fifth Central Hospital of Tianjin: L. Chen; Tianjin Medical University Central General Hospital: R. Jiang; The People’s Hospital of Heijian : Y. Ma; Nanhe County People’s Hospital: J. Yao; The No.1 Hospital of Shijiazhuang: Q. Jiao; The First People’s Hospital of Yuanping: X. Xin; Yuxian People’s Hospital: SD. Guo; The First Hospital of Shanxi University: H. Wang; Shanxi Dayi Hospital: H. Tong; The First Affiliated Hospital of Baotou Medical College: L. Wu; People's Hospital of Ordos Dongsheng District: J. Zhang; Hebei General Hospital:B. Pan; Liaoning Thrombus Treatment Centre of Integrated Chinese and Western Medicine : G. Tang; China-Japan Union Hospital of Jilin University: X. Fang; Tieling Central Hospital: D. Zhao; Tiemei General Hospital of Liaoning Province Health Industry Group: DH. ZhaoSun; The First People’s Hospital of Shenyang: Xin. Li; Huashan Hospital of Fudan University Neurosurgery Department: J. Hu; Xianhua Hospital of Shanghai JiaoTong University School of Medicine: M. Chen; The Second Affiliated Hospital of Suzhou University: Y. Cao; The Frist Affiliated Hospital of Suzhou University: Z. Wang; Xuzhou Central Hospital: D. Jiang; Xinhua Hospital of Zhejiang Province: B. Zhou; Jinhua Municipal Central Hospital: G. Zhou; Zhongshan Hospital Affiliated to Xiamen University: Q. Yang; Yantai Yu Huang Ding Hospital: X. Wu; Qingdao Municipal Hospital: W. Wang; Liaocheng People's Hospital: J. Wang; Dezhou People's Hospital: L. Shi; Dong'e People’s Hospital: W. Zhang; ShanDong Provincial QianFoshan Hospital: X. Meng; Linyi People’s Hospital: F. Meng; Huashan Hospital of Fudan University Neurology Department: Y. Tang; Jiangsu Rudong County People's Hospital: X. She; Zhoushan Hospital: H. Wang; The 904th Hospital of the Joint Logistics Support Force of the Chinese People's Liberation Army:D. Shi; Zhangjiagang First People's Hospital: H. Liu; Xinghua Traditional Medical Chinese Hospital: B. Tang; The First Affiliated Hospital of Zhengzhou University: F. Guo; Dancheng County People’s Hospital: Y. Yang; The 91st Central Hospital of the Chinese People's Liberation Army: P. Zhang; Zhongnan Hospital of Wuhan University: J. Chen; Renmin Hospital of Wuhan University: Q. Cai; Union Hospital Tongji Medical College of Huazhong University of Science and Technology: R. Fu; Tongji Hospital Tongji Medical College of Huazhong University of Science and Technology: T. Lei; Wuhan Central Hospital: J. Li; Wuhan Third Hospital: X. Yu; Wuhan First Hospital: F. Duan; Jingzhou Central Hospital: T. Chen; Guangzhou First People’s Hospital: Z. Cao; The 987th Hospital of the Joint Logistics Support Force of the Chinese People's Liberation ArmyThe Third Hospital of Chinese People's Liberation Army: X. Wang; No.215 Hospital of Shanxi Nuclear Industry: Y. Zuo; People's Hospital of Xinjiang Uygur Autonomous Region: X. Yang. Chile- Clínica Alemana de Santiago: P. Muñoz Venturelli ; Complejo Asistencial Dr. Victor Ríos Ruiz: C. Figueroa; Hospital Base San José de Osorno: N. Conejan; Hospital Clinico Chillán Herminda Martin: A. Rojo; Hospital Dr. Luis Tisné Brousse: R. Guerrero; Hospital La Florida Dra. Eloísa Díaz: J. Vallejos; Hospital Carlos Van Buren: J. Gigoux. Peru- Instituto Nacional de Ciencias Neurológicas: C. Abanto; Mexico- Instituto Nacional de Neurología y Neurocirugía: A. Arauz; Brazil- Hospital de Clinicas de Porto Alegre : S. Ouriques Martins ;Clinica Neurologica e Neurocirurgica de Joinville Ltda : C. Cabral; Hospital das Clinicas da Faculdade de Medicina de Riberao Preto da Universidade de Sao Paulo: O. Pontes ; Faculdade de Medicina de Botucatu/UNESP: R. Bazan ; Hospital Moinhos de Vento : G. Weiss. Vietnam- People Hospital 115: H. Nguyen; Nguyen Tri Phuong Hospital: D. Vo; University Medical Center Ho Chi Minh City: B. Nguyen; Bach Mai Hospital: D. Mai;103 Military Hospital: D. Pham; Thong Nhat Hospital: T, Nguyen. Nigeria- University of Ilorin Teaching Hospital, Ilorin: K. Wahab; Ahmadu Bello University Teaching Hospital, Zaria: R. Obiako; Lagos University Teaching Hospital, Lagos: N. Okubadejo. India- Christian Medical College and Hospital, Ludhiana: J. Duri Pandia; Guru Gobind Singh Hospital, Faridkot: S. Singh; Baptist Christian Hospital, Assam: J. Webater; Shree krishna Hospital, Gujarat: S. Desai; KLES Dr. Prabhakar Kore Hospital & MRC: A. Saroja; GNRC Hospital Dispur: R. Das; Government Medical College, Thiruvananthapuram: T. Iype; St. Stephen's Hospital, Delhi: I. Sebastian; Banaras Hindu University, Varanasi: A. Pathak; Government Medical College, Jammu: A. Shah. Pakistan- The Aga Khan University Karachi: M. Wasay; South City Hospital Karachi: R. Shanker; Shifa International Hospital, Islamabad: R. Shoaib. Si Lanka- National Hospital Sri Lanka: B. Senanayaka; Colombo South Teaching Hospital: V. Rajendiran; Jaffna Teaching Hospital: A. Keshavaraj; Karapitiya Teaching Hospital: A. Dissanayake; Kandy National Hospital: I. Wijeweera; District General Hospital Gampaha: N.Fernando; Kurunegala Teaching Hospital: D.Palliyaguruge.
