# Supplementary material for: INTEnsive care bundle with blood pressure reduction in acute cerebral hemorrhage trial (INTERACT3): study protocol for a pragmatic stepped-wedge cluster-randomized controlled trial
Source: Trials. 2021 Dec 20;22:943. doi: 10.1186/s13063-021-05881-7 (PMC8686093; doi:10.1186/s13063-021-05881-7)
Supplement: Supplementary file 3 — Additional file 3. Informed consent materials. [file 13063_2021_5881_MOESM3_ESM.pdf]

[Insert Hospital name/logo]

HOSPITAL INFORMATION & CONSENT FORM

What we know about stroke due to intracerebral hemorrhage

Acute intracerebral hemorrhage (ICH) is a serious form of stroke that is due to the spontaneous rupture of blood vessel in the brain. ICH is usually caused by longstanding high blood pressure, or hypertension, and it affects several million people in the world each year. Patients who experience ICH are at high risk of death, most in the first few days after onset from pressure due to the expanding blood clot in the brain. Patients who survive the illness are often left with some degree of physical disability and memory loss. They are also at high risk of recurrent ICH as well as other serious types of cardiovascular disease that are due to blockage of blood vessels in the brain (ischaemic stroke) and heart (heart attack).

What we don't know about the treatment for intracerebral hemorrhage

Unfortunately, knowledge of the best strategies to manage patients with ICH is limited. Surgery to remove or reduce the clot of blood in the brain is often done in those patients with more severe types of ICH. However, surgery has risks and doctors are often uncertain about which patients are likely to gain the most benefit from this treatment with minimal harms. In regard to the overall care of patients with ICH, this is primarily supportive, that means doctors and nurses are monitor patient's vital functions (breathing, blood pressure, and circulation), effects of the brain injury (neurological function, brain swelling), aim to recognise quickly and effectively treat any complications relate to being immobile in bed (blood clots in the legs) and infections (pneumonia, urinary tract), and promote therapy to assist recovery and rehabilitation. There is some evidence indicating that good control of blood pressure and some other abnormalities of the circulation can improve recovery. However, the evidence is not strong enough to develop policies and there is still uncertainty about the benefits of improving the circulation in relation to the timing and intensity of treatment in certain types of patients, for example, those with severe type of ICH.

What is involved in this research

This research study is called the third INTEnsive care bundle with blood pressure Reduction in Acute Cerebral haemorrhage Trial (**INTERACT3**). It has been designed to determine whether the implementation of training and information to doctors about - the control of blood pressure and elevated sugar in the blood, the early treatment of fever, and the correction of any abnormality of clotting in the blood related to anticoagulation medication – will improve recovery for patients with ICH. While these treatments are already being undertaken as part of routine care in hospitals around the world, not every patient receives them to the same extent, and there is considerable variation in timing and intensity of the treatments in patients within and between hospitals.

This research aims to determine whether standardising the use of treatments to control physiological parameters in the circulation for most patients with ICH who are admitted to hospital will benefit them in terms of surviving with better levels of recovery several months later.

The study is being conducted across nearly 110 hospitals, most of them located in China, but also in several other countries including Chile, Peru, Mexico, Brazil, Vietnam, Pakistan, Nigeria and Iraq. The protocols and training of hospital staff will be rolled out in staged, randomized, manner across these hospitals. The hospitals will commence collecting data on the routine management of patients with ICH as soon as they receive ethics approval but will be informed at varying time points

about participating in the intervention 'implementation phase' of training and use of protocols. Patients will not notice any difference in their care as the research activity is being undertaken separately, in the background to their care.

All ICH patients who present to the emergency department or other department will be included in the study. They are provided with a Patient Information Form and Consent Form for their approval to participate in the research to: (i) allow release of some of their medical information to be used for research purposes, and (ii) to agree to receive a telephone call from a research person associated with the study to assess your health in about 6 months after their admission to hospital. In order for this research person to be able to contact the patient (or your family or doctor), they are required to agree to send their contact details, including mobile telephone number, to the research office that organizes the follow-up assessments.

As the study involves coordinated implementation of routine aspects of care, and non-regulatory approved new drugs or treatments, this Hospital Consent Form is to allow the study to proceed smoothly without interruption for the randomized intervention of the care bundle of protocols to be implemented in patients without their consent. Specifically, the hospital is responsible for:

(i) acting as an advocate in the best interest of ICH patients;
(ii) approving the use of the goal-directed care bundle described in the protocol becoming a standard medical management during randomized intervention phase; and
(iii) approving adherence to the protocol during the study period.

The Hospital Consent Form is to be signed before commencement of the study at a hospital site. Participation of patients in this study is entirely voluntary. They are in no way obliged to participate and they can withdraw at any time without giving a reason. Whatever they decide, please be assured that it will not affect their relationship with the hospital staff involved in this research.

The study is a collaboration between The George Institute for Global Health (Australia) Beijing Representative Office (Chief Investigator, Professor Craig ANDERSON) and the Department of Neurosurgery of West China Hospital (Co-Chief Investigator, Professor YOU Chao).

All aspects of the study, including the results, will be kept strictly confidential, and individual patients, hospital or staff, will not be identified in any publication. .

This study has been approved by the Ethics Review Committee (insert name of ethics committee). Any person with concerns or complaints about the conduct of this study should contact the following people:

The Regional Coordinating Centre on (insert telephone number) and quote local protocol number ……………………………………. or the National Leader:

Name: insert contact details

Contact Number: insert contact details

Address: insert contact details

[Insert Hospital name/logo]

CLUSTER GUARDIAN CONSENT FORM

[Hospital name]...

[Address]...

I have read and understood the information provided in the Hospital Information Sheet. Any questions I have asked have been answered to my satisfaction. I agree to participate in the INTERACT3 study, with the understanding that I can withdraw from the study at any time without compromise to my relationship with the researchers. I understand all information will remain strictly confidential. I agree that research data collected for the study may be published or may be provided to other researchers in a form that does not identify patients nor my hospital in any way.

By signing my name, I indicate agreement to:

(i) act on behalf of the best interests of patients with ICH to be included in the study;

(ii) approve the goal-directed care bundle described in the protocol becoming standard medical treatment during the randomized intervention phase for departments; and

(iii) the hospital will adhere to the intervention and protocols during the study period.

Name:...

(Please print name using block letters)

Position: ....

Signature: ....

Date: ....

Daytime Telephone Number: ....

[Insert Hospital Name /logo]

INTERACT3 (**INTE**nsive care bundle with blood pressure **R**eduction in **A**cute **C**erebral haemorrhage **T**rial)

PATIENT INFORMATION & CONSENT FORM

Introduction

The hospital is currently participating in a research study to investigate the effect of organized management involving control of blood pressure, elevated blood sugar, treatment of fever (or pyrexia), and correction of blood coagulation ('thinning of blood clotting') in patients with stroke due to acute intracerebral hemorrhage (ICH), which is bleeding in the brain. Although guidelines recommend early treatment of these abnormal physiological parameters, there is uncertainty over the timing and intensity to treatment, and in which patients have the most (or least) to benefit, if at all. This research aims to assess the use of training and information provision to doctors to improve the use of treatments as part of an 'early intensive care bundle' as part of routine clinical practice.

This study is being conducted in many hospitals around the world as part of an international cooperation coordinated by The George Institute for Global Health (Australia) Beijing Representative Office and the Department of Neurosurgery of West China Hospital in Chengdu, China.

In this hospital, these people are responsible for the study:

____

____

____

[names, positions, departments].

Please read the following information and ask your doctor any questions you may have.

What does participation in this study involves?

Participation in the study involves:

- Some aspects of your medical information will be recorded for the study after you are admitted to the hospital.
- Information on some aspects of your medical care and management during your stay in the hospital will be used in the study.
- You may receive a goal-directed treatment of your blood pressure, circulation, elevated temperature and correction of abnormally high blood sugar. In addition,

you will receive usual standard of care nursing and medical stroke management, information and procedures as is routine in hospital.

- Your personal identifiable information (include name and contact number) will be recorded for study follow-up. At 6 months (±1 month), you will be contacted for a telephone interview by a follow-up staff person to evaluate the state of your health and level of recovery. This will take about 20 minutes.
- The use of your medical information will be grouped with other patients, will be used for statistical analysis and scientific reports. This information is analysed by groups of people and without any personal identifiable information.

Are there any risks or benefits?

For INTERACT3 study, all of their management are within the range of routine clinical practice and several guideline recommendation. The risk of serious reactions are small. However, all medical procedures – whether for diagnosis or treatment, routine or experimental – involve some risk of injury from drugs or procedures.

We recognise that there may be some risks associated with this study that are presently unknown and unforeseeable, but we believe these to be very small as we are study standardisation of management procedures that are already being undertaken in routine clinical practice all over the world. In spite of all precautions, you may develop medical complications whilst participating.

Benefits

While we intend for this study to advance medical knowledge and improve treatment of patients with ICH in the future, it may not directly benefit the person you represent

Confidentiality

All the information collected from you for this research study will be treated in strict confidence. Only the INTERACT3 researchers, study monitors and representatives of regulatory authorities and the ethics committee, may have direct access to your medical records. Access to medical records is required to check the accuracy of the information collected and to ensure that this study is being carried out according to local requirements and regulatory guidelines.

All information collected from your medical records for the study will be stored electronically in a specifically designed and secure database at The George Institute for Global Health, Sydney, Australia. Your information will be identified only by your initials, date of birth and a study registration number. Any information transferred electronically will be coded to protect your confidentiality. All computer records will be password protected. Personal identifiable information (name, contact number etc.) will be collected and stored separately to other collected data, and responsible by specific personnel. The use of personal identifiable information will be stringently controlled and recorded. Due to the study request, your indefinable information will be forwarded to the third party company for study follow-up. We have agreement with the third party company to clarify the responsibilities and obligators; and we will supervise the company to ensure the security of your personal identifiable information.

The study results analyzed from unidentifiable data may be presented at conferences or in scientific publications. These unidentifiable data might be shared to other researchers around the world according to the high-standard data sharing policy in the future. The data will be stored and used following the stringent standard of data safety. Your identity will not be exposed.

You have a right of access to, and to request correction of, information held about you in accordance with local laws. Please contact one of the researchers named below if you would like to access your information.

Complications

If you suffer any injuries or complications as a result of this study, you should contact the study doctor as soon as possible, who will assist you in arranging appropriate medical treatment.

In addition, you may have a right to take legal action to obtain compensation for any injuries or complications resulting from the study. Compensation may be available if your injury or complication is sufficiently serious and related to the study.

Will taking part in this study cost me anything?

Participation in this study will not cost you anything and there will not be any payment for participating.

Your participation is voluntary

Participation in this study is entirely voluntary. You do not have to take part in the study. If you do take part, you can withdraw at any time during the study without having to give a reason. Whatever your decision, please be assured that it will not affect your medical treatment or your relationship with the staff who are caring for you.

Contact Details

When you have read this information, your treating doctor will be available to discuss and answer any questions you may have. If you would like to speak with someone at any stage, please feel free to contact the Principal Investigator at this hospital on [INSERT CONTACT NUMBER] or the following person:

Name: [INSERT NAME OF STUDY COORDINATOR OR KEY RESPONSIBLE PERSON]

Role: [INSERT ROLE OF KEY RESPONSIBLE PERSON]

Telephone: [INSERT TELEPHONE NUMBER OF KEY RESPONSIBLE PERSON]

Ethics approval and Conplaints

This study has been approved by the [INSERT NAME OF ETHICS COMMITTEE]. Any person with concerns or complaints about the conduct of this study should contact the Ethics Committee on [INSERT TELEPHONE NUMBER OF ETHICS COMMITTEE].

This information sheet is for you to keep.

[Insert Hospital Name /logo]

PATIENT CONSENT FORM

……………………………………………………………………………………………………….. ....

(address)

I have read and understood the Patient Information Sheet (page 1-4 of this document) regarding the **INTERACT3** research study, and have been inform about the collection of information, and 6 months follow-up process.

I have been made aware of the procedures involved in the study, including any known or expected inconvenience, risk, discomfort or potential side effect as far as they are currently known by the researchers. I have had an opportunity to ask questions and I am satisfied with the answers I have received. I also understand that my participation in the study allow the researchers and others, as stated in the patient information sheet to have access to my medical records for the purpose of the study.

I also agree to participate in the telephone interview to evaluate the state of my health and level of recovery.

I freely choose to participate in this study and understand that I can withdraw at any time.

- **INTERACT3** ***YES/ NO*** *(please circle)*

Patient/Patient Responsible Name (Print) ……………………………………………..………………………

Relationship with patient ……………………………………………………………………..……………………………

Signature…………………………………………………………………………………… Date……………………………………

Name of person obtaining the consent (Print)…………………………………………………………………………

Signature……………………………………………………………………………………Date……………………………………

Name of witness (if applicable)..…

Signature (if applicable)……………………………………………………… Date.........................……………

INTERACT3 Consent Withdrawal Form

Study name	**INTE**nsive care bundle with blood pressure **R**eduction in **A**cute **C**erebral haemorrhage **T**rial（INTERACT3）
Site name	
Patient/Patient Responsible Name (Print)	
Reason of withdrawal	
If agree continue to use the data have been collected	☐ Yes ☐ No
Withdrawal style	☐ Self-pick-up<br>☐ Post<br>Post address :
Patient/Patient Responsible Name (signature)	
Patient/Patient Responsible contact number	
Investigator name(signature)	
Investigator contact number	
