# Supplementary material for: INTEnsive care bundle with blood pressure reduction in acute cerebral hemorrhage trial (INTERACT3): study protocol for a pragmatic stepped-wedge cluster-randomized controlled trial
Source: Trials. 2021 Dec 20;22:943. doi: 10.1186/s13063-021-05881-7 (PMC8686093; doi:10.1186/s13063-021-05881-7)
Supplement: Supplementary file 4 — Additional file 4. TSC Charter. [file 13063_2021_5881_MOESM4_ESM.pdf]

The George Institute for Global Health

INTEnsive care bundle with blood pressure Reduction in Acute Cerebral haemorrhage Trial

TRIAL STEERING COMMITTEE (TSC) CHARTER

Chair, Independent Steering Committee: Professor Thompson Robinson (University of Leicester, UK)

Principal Investigator: Professor Craig Anderson (The George Institute for Global Health, Beijing Representitive Office, China; Faculty of Medicine, University of New South Wales, Australia)

Co-Principal Investigator: Professor Chao You (West China Hospital, Sichuan University, China)

Chair, Data Safety Monitoring Board: Professor Robert Herbert (Neuroscience Research Australia; School of Medical Sciences, University of New South Wales, Australia)

ClinicalTrials.gov: NCT03209258

Chinese Trial Registry: ChiCTR-IOC-17011787

Trial Sponsor: The George Institute for Global Health, Beijing Representitive Office

Date of Document:

Version 1.0: 30 July 2020

Table of Contents

1. Introduction....1

2. Role & Responsibilities of the Steering Committee....2

3. Steering Committee Organisation....4

3.1 Selection of Steering Committee Members....4

3.2 Qualifications of Steering Committee Members....4

3.3 Training of the Steering Committee....4

3.4 Steering Committee Chair & Deputy Chair....4

3.5 Steering Committee Members....4

4. Steering Committee Meetings....5

4.1 Schedule of Meetings....5

4.2 Attendance....5

4.3 Declaration of Conflict of Interest....6

4.4 Confidentiality....6

4.5 Copyright....6

4.6 Format of Meetings....6

4.7 Meeting Minutes....6

5. Membership....7

6. Version Control....8

7. Appendix 1 – Steering Committee Charter Signature Sheet....9

1. Introduction

INTERACT3 is an investigator-initiated and conducted, international, multicentre, prospective, stepped-wedge, cluster randomised, blinded outcome assessed study to determine the effectiveness of a goal-directed care bundle of active management involving early physiological control (intensive blood pressure [BP] lowering, glycaemic control, and early treatment of pyrexia) and reversal of anticoagulation, versus usual standard of care, on functional outcome in patients with acute stroke due to acute spontaneous intracerebral haemorrhage (ICH).

All patients who meet the eligibility criteria for the study will be randomised to either:

Usual care group: For patients in the usual-care group, decisions about the location of care delivery, investigations, monitoring, and all treatments will be made by the treating clinical team.
Interventional group: A goal-directed care bundle that involves the rapid correction (<1 hour) of physiological variables as soon as the abnormality is recognised and for the control to be maintained in patients for 7 days or hospital discharge (or death, if sooner):

1) intensive BP lowering to systolic target of <140 mmHg;
2) glucose control target 6.1-7.8 mmol/l for non-diabetic; 7.8-10.0 mmol/l for diabetic patients;
3) treatment of pyrexia to a target body temperature ≤37.5 °C;
4) reversal of anticoagulation to target INR <1.5 involving use of vitamin K and prothrombin complex concentrate (PCC), or alternatively fresh frozen plasma (FFP).

The Primary Outcome is an assessment of the full range of scores on the 7-level, modified Rankin scale (mRS), measured at 6 months after patients are admitted to hospital. Secondary Outcomes include early neurological recovery according to the National Institute of Health stroke scale (NIHSS) assessed at 7 days, poor outcome (defined by mRS scores 3-6), health-related quality of life (HRQoL) on the EuroQoL Group 5-Dimension self-report questionnaire (EQ-5D), residence at 6 months, duration of initial hospitalisation, and further hospital admission.

This Charter will define the primary responsibilities of the Trial Steering Committee (TSC), its relationship with other trial committees, membership, and purpose and timing of meetings. The Charter will also provide the procedures for selecting TSC members and organising TSC meetings.

2. Role & Responsibilities of the TSC

The TSC is responsible for the execution of the study design, protocol, data collection and analysis plan, as well as publications. Furthermore, the TSC:

- is the decision-making body responsible for implementing modifications to the protocol, including those that may result from recommendations from the study's Data Safety Monitoring Board (DSMB), as outlined in the INTERACT3 DSMB Charter;
- has the right to appoint new members and co-opt others to add to the integrity of the conduct of the study and analyses;
- includes leading scientific experts to be involved in the development, approval, and maintenance of the TSC Charter;
- will monitor and supervise study progress towards its interim and overall objectives;

- will review at regular intervals relevant information from other sources (e.g. other related trials);
- will report to the Sponsor on progress of the trial, and if necessary, to MRC as the funder;
- advise the Chief Investigator, Sponsor and MRC (as funder) on publicity and presentation of all aspects of the trial;
- may also be involved in study site selection and education, for the investigators and staff, on an as needed basis.

3. TSC Organisation

3.1 Selection of TSC Members

The Chair and Deputy Chair of the TSC will decide the final membership of the TSC as well as any changes to the membership for the duration of the INTERACT3 study. This membership should also be submitted to MRC to get approval. Membership is for the duration of the trial unless the member withdraws their membership or is deemed by the TSC Chair and Deputy Chair to be unable to fulfil his/her responsibilities. These responsibilities include, but are not limited to, overviewing all aspects of the study design, protocol development, conduct, analysis and publication.

3.2 Qualifications of TSC Members

Members will be healthcare providers or other relevant individuals, statisticians, or epidemiologists with clinical and/or research expertise relevant to the design and conduct of the INTERACT3 study. Documentation of the qualifications will be maintained by The George Institute in the form of curricula vitae for the selected TSC members.

3.3 Training of the TSC Committee

Members of the TSC will be provided with training on:

- Orientation to the project
- Specific committee objectives

This training will be documented and the details kept centrally at The George Institute.

3.4 TSC Committee Chair & Deputy Chair

The specific responsibilities of the TSC Chair and Deputy Chair include:

- To preside over TSC meetings or delegate to an appropriate designee from the TSC
- To facilitate the finalisation and sign-off of the TSC Charter and associated documents
- To ensure that an accurate account of TSC conference calls and meetings is made and circulated to all TSC members along with relevant representatives from the operational team
- To ensure that all decisions made by the committee are communicated to all TSC members
- To ensure that high quality is maintained in all aspects of study design, protocol development, conduct, analysis and publication
- To communicate to the DSMB Chair any decisions made or issues found

3.5 TSC Committee Members

The TSC members are responsible for the following:

- To participate in all TSC teleconferences and meetings

- To participate in discussions related to all aspects of study design, protocol development, conduct, analysis and publication
- To communicate to the TSC Chair and Deputy Chair any schedule conflicts, including extended time away from office, which may impact on their ability to be part of the TSC
- To provide input to the TSC Chair and Deputy Chair regarding specific issues that may need to be addressed by the DSMB
- To ensure that all materials and information are kept strictly confidential and are not discussed or disclosed with anyone external to the TSC unless specifically authorised in this charter.

Responsibilities of individual members:

- Each individual member is expected to assist in achieving the functions of the project through active contributions. In discussing issues, members will bring their expertise, decisiveness; focus on the future and good humor to bear
- Undertake activity as a representative of their agency/country for the project
- Promote and provide advocacy for the project
- All members of the Committee are required to declare any conflict of interest at all times
- During TSC Committee membership, the member will not reveal any confidential or proprietary information entrusted in the course of their duties
- Upon cessation of membership, and thereafter, the member shall not reveal any confidential or proprietary information, which they obtained while a member of the committee, and may not use or retain, or attempt to use or retain, any such information, documents or data
- During membership, and thereafter, the member will respect the copyright of any information and resources developed under the auspices of the committee as agreed by the full membership

4. TSC Committee Meetings

4.1 Schedule of Meetings

TSC meetings will be held twice annually, and near the time (following) DSMB meetings and in accordance with the schedule below. The TSC Chair and Deputy Chair will have the right to determine the need for additional meetings on an as needed basis:

**Face to face meeting (where feasible)**	Once a year, opportunistically held in accordance with the location and timing of the European Stroke Organisation Conference in May or the World Stroke Congress in October of any given year.
**Teleconference**	Once a year, either in May or October, timed alternately with the face to face meeting
**Other meetings**	Convened as required

4.2 Attendance

- To ensure the ongoing management of core business there is an expectation that TSC Committee members will attend all meetings

- Where this is not possible a proxy from the organisation may be nominated to attend
- A quorum is set at 50% of current membership
- Observers will be invited to attend from the following representatitves: Funder(MRC), Host Institution, Sponsor.
- National leaders and other people outside the TSC Committee can be invited to participate in particular discussions on relevant issues by agreement of the TSC Committee.
- Members are required to inform the project manger of their non-attendance at any meeting and notify their nominated proxy prior to the meeting date

4.3 Declaration of Conflict of Interest

All TSC members are required to declare any conflict of interest at the beginning of each TSC meeting, during the trial and in analysis of results.

4.4 Confidentiality

During TSC membership, all members will not reveal any confidential or proprietary information entrusted in the course of their duties. Upon cessation of membership, and thereafter, the member shall not reveal any confidential or proprietary information which they obtained while a member of the committee, and may not use or retain, or attempt to use or retain, any such information, documents or data.

4.5 Copyright

During membership, and thereafter, all information and resources developed under the auspices of the committee shall remain the property of the sponsor.

4.6 Format of Meetings

Prior to each meeting, an agenda and relevant documentation will be circulated to all TSC members and other individuals attending the meeting in a non-voting capacity.
The agenda will usually follow the format below:

- Introductions
- Declaration of any conflicts of interest
- Review agenda
- Minutes from last meeting
- Review of actions arising from previous TSC meetings
- Overall study status, including site status, recruitment, adherence, serious adverse events, protocol violations, operational issues
- Report from the DSMB (if relevant)
- Consideration of other items relevant to the study
- Review and summarise new actions from the current meeting
- Any other business
- Plan, date and location for next meeting

4.7 Meeting Minutes

Meeting minutes will be taken by the Project Manager or delegate at each meeting. The minutes will be sent to the Chair and Deputy Chair, for review after a meeting and prior to circulation among the remaining attendants. Following review by the chairs, all TSC members with have the opportunity to comment on the minutes within the timeframe of two weeks, after which, the minutes will be finalised. Finalised minutes will be presented for acceptance at the next meeting, report to funder (MRC) and filed at The George Institute.

5. Membership

5.1 Term of Membership

- Independent TSC members will be invited whose organisation are not taking part in this trial
- Members of the TSC Committee will comprise individuals interested in the operations and success of the project
- The committee will have representation from low-middle income country regions participating in the project
- Membership will be for the duration of the trial
- If any member leaves during the course of the trial, the Operations Executive Committee will appoint an appropriate replacement.

5.2 TSC Members

Name	Role	Country	Contact details
Professor Thompson (Tom) Robinson (Chair)	Independent member	UK	University of Leicester<br>T: +44 (0)116 204 4752<br>E: tgr2@leicester.ac.uk
Professor J Jaime Miranda (Deputy Chair)	Independent member, LMIC representative	Peru	CRONICAS Centro de Excelencia en Enfermedades Crónicas, Universidad Peruana Cayetano Heredia, Av. Armendáriz 497, Miraflores, Lima 18, Perú<br>T: (+51) 1 241 6978<br>E: jaime.miranda@upch.pe
Professor Craig Anderson	Co-Principal Investigator	China	The George Institute for Global Health, Beijing Representitave Office<br>T +86 10 82800577 ext 557<br>M +86 15210347573<br>E: canderson@georgeinstitute.org.cn
Professor Chao You	Co-Principal Investigator	China	West China Hospital<br>T: +86-28-85422023<br>E: youchao@vip.126.com
Dr Lili Song	Associate investigator, Global Project lead	China	The George Institute for Global Health, Beijing Representitave Office<br>M +86 13916466400

			E: lsong@georgeinstitute.org.cn
Dr Adrian Parry-Jones	Independent member	UK	University of Manchester<br>E:adrian.parry-jones@manchester.ac.uk
Professor Nikola Sprigg	Independent member	UK	University of Nottingham<br>T: +44 (0) 115 823 1778<br>E: Nikola.Sprigg@nottingham.ac.uk
Ms Sophie Durrans	Observer	UK	Department of Health and Social Care<br>E: Sophie.Durrans@dhsc.gov.uk
Ann Bamford	Patient representative	UK	E: nabamford@btinternet.com
Olivia Smith	Patient representative	UK	E: livvysmith@hotmail.com

5.3 National leaders

Name	Country	Contact details
Professor Octavio Marques Pontes-Neto	Brazil	University of São Paulo<br>T: +5516-36021202<br>E: opontesneto@fmrp.usp.br
Dr. Paula Munoz Venturelli	Chile	Universidad del Desarrollo<br>E: pmventurelli@gmail.com
Professor Antonio Arauz	Mexico	The National Institute of Neurology in Mexico City<br>E: arauz@prodigy.net.mx
Professor Kolawole Wahab	Nigeria	University of Ilorin<br>T: +234 803 380 0873<br>E: wahab.kw@unilorin.edu.ng
Professor Mohammad Wasay	Pakistan	Aga Khan University<br>T: (9221) 493 0051<br>E: mohammad.wasay@aki.edu
Dr. Carlos Abanto	Peru	National Institute of Neurological Sciences, Lima<br>T: (+51) 9985-43158<br>E: carlosabantoa@yahoo.com
Dr Nguyen Huy Thang	Vietnam	Stroke Unit, 115 Hospital, Ho Chi Minh city<br>T: +84 9831 11866<br>E: dr_nguyenhuythang@yahoo.com

6. Version Control

Version	Date Finalised	Changes Made
1.0	30 July 2020	Original

Appendix 1 – Steering Committee Charter Signature Sheet

Members of the Steering Committee undertake their respective roles on the Steering Committee in a volunteer capacity. By signing below, all members agree to comply with the requirements set forth within this Charter and in doing so shall be indemnified by The George Institute.

I have read and approve this Charter:

Name (print)

Professor Thompson Robinson

Signature

T.G.R

Date of Signature (dd/mmm/yyyy)
24/11/2020

Appendix 1 – Steering Committee Charter Signature Sheet

Members of the Steering Committee undertake their respective roles on the Steering Committee in a volunteer capacity. By signing below, all members agree to comply with the requirements set forth within this Charter and in doing so shall be indemnified by The George Institute.

I have read and approve this Charter:

Name (print) J. Jaime Miranda

Signature

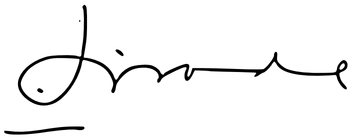

Date of Signature (dd/mmm/yyyy) 10 December 2020

Appendix 1 – Steering Committee Charter Signature Sheet

Members of the Steering Committee undertake their respective roles on the Steering Committee in a volunteer capacity. By signing below, all members agree to comply with the requirements set forth within this Charter and in doing so shall be indemnified by The George Institute.

I have read and approve this Charter:

Name (print)

Professor Craig Anderson

Signature

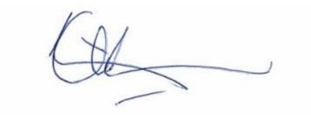

Date of Signature (dd/mmm/yyyy)
24/11/2020

Appendix 1 – Steering Committee Charter Signature Sheet

Members of the Steering Committee undertake their respective roles on the Steering Committee in a volunteer capacity. By signing below, all members agree to comply with the requirements set forth within this Charter and in doing so shall be indemnified by The George Institute.

I have read and approve this Charter:

Name (print) Chao You

Signature Chao You

Date of Signature (dd/mmm/yyyy) 15/12/2020

Appendix 1 – Steering Committee Charter Signature Sheet

Members of the Steering Committee undertake their respective roles on the Steering Committee in a volunteer capacity. By signing below, all members agree to comply with the requirements set forth within this Charter and in doing so shall be indemnified by The George Institute.

I have read and approve this Charter:

Name (print) Lili Song

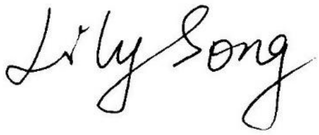

Signature

Date of Signature (dd/mmm/yyyy) 10/12/2020

Appendix 1 – Steering Committee Charter Signature Sheet

Members of the Steering Committee undertake their respective roles on the Steering Committee in a volunteer capacity. By signing below, all members agree to comply with the requirements set forth within this Charter and in doing so shall be indemnified by The George Institute.

I have read and approve this Charter:

Name (print) Adrian Parry-Jones

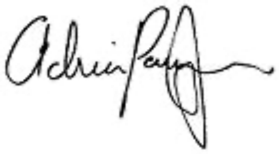

Signature

Date of Signature (dd/mmm/yyyy) 24 NOV 2020

Appendix 1 – Steering Committee Charter Signature Sheet

Members of the Steering Committee undertake their respective roles on the Steering Committee in a volunteer capacity. By signing below, all members agree to comply with the requirements set forth within this Charter and in doing so shall be indemnified by The George Institute.

I have read and approve this Charter:

NIKOLA SPRIGG

Name (print)

Nikola Sprigg

DR. NIKOLA SPRIGG.

Signature

04/Dec/2020

Date of Signature (dd/mmm/yyyy)

Appendix 1 – Steering Committee Charter Signature Sheet

Members of the Steering Committee undertake their respective roles on the Steering Committee in a volunteer capacity. By signing below, all members agree to comply with the requirements set forth within this Charter and in doing so shall be indemnified by The George Institute.

I have read and approve this Charter:

Name

Ann Bamford

Signature

Date of Signature (07/12/2020)

Appendix 1 – Steering Committee Charter Signature Sheet

Members of the Steering Committee undertake their respective roles on the Steering Committee in a volunteer capacity. By signing below, all members agree to comply with the requirements set forth within this Charter and in doing so shall be indemnified by The George Institute.

I have read and approve this Charter: YES

Name (print) OLIVIA SMITH

Signature 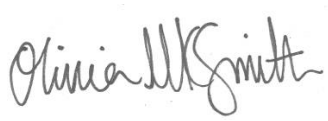

Date of Signature (dd/mmm/yyyy) 7/12/2020
